# Nicotinamide Reverses the Warburg Effect in CHO Cell Culture

**DOI:** 10.1002/bit.70224

**Published:** 2026-04-29

**Authors:** James Morrissey, Ayca Cankorur‐Cetinkaya, Luigi Grassi, Annie J. Harwood‐Stamper, Jonathan Welsh, Ryte Poskute, Kasia Kozakowska‐McDonnell, Cleo Kontoravdi

**Affiliations:** ^1^ Department of Chemical Engineering Imperial College London London UK; ^2^ Cell Culture and Fermentation Sciences, Biopharmaceuticals R&D, AstraZeneca Cambridge UK; ^3^ Analytical Sciences, Biopharmaceuticals R&D, AstraZeneca Cambridge UK

## Abstract

The Warburg effect, the preferential conversion of glucose‐derived pyruvat to lactate despite available oxygen, is a key feature of Chinese hamster ovary (CHO) cell culture. Lactate accumulation in recombinant protein‐producing cell culture is an inefficient usage of glucose, as well as being deleterious to cells. Lactate accumulation lowers culture pH, requiring base addition to maintain bioreactor pH setpoint, which subsequently leads to hyperosmolarity, adversely impacting cell growth, productivity and product quality. A key driver for the Warburg effect, and hence lactate accumulation, is the need to regenerate NAD^+^ consumed during glycolysis. Since oxidative phosphorylation has limited capacity to recycle NADH back to NAD^+^ at high glycolytic fluxes, cells rely on lactate dehydrogenase (LDH) to convert pyruvat to lactate, simultaneously regenerating NAD^+^ and sustaining glycolysis. Thus, providing the cells' capacity to generate more NAD^+^ would decrease the reliance on the Warburg effect. In this study, feeding the NAD^+^ precursor nicotinamide (NAM) leads to reversal of the Warburg effect, inducing the “lactate shift” 3 days earlier in cell culture and reducing peak lactate concentration by 40%. Transcriptomic analysis further confirms this metabolic shift, with an upregulation of key mitochondrial electron transport chain genes. NAM supplementation increased Fc galactosylation without affecting fucosylation, sialylation, or high‐mannose species. These results identify NAD^+^/NADH balance as a key regulator of the Warburg effect and demonstrate NAM supplementation as a simple, cost‐effective strategy to mitigate lactate accumulation and improve metabolic efficiency in CHO cell cultures.

## 1 Introduction

Chinese hamster ovary (CHO) cells are the workhorse of biopharmaceutical production (Walsh and Walsh 2022). CHO cells offer several advantages, including the capacity for post‐translational modifications similar to those in human cells, which is crucial for the efficacy and safety of biopharmaceutical products. Over the years, CHO cells have been optimised for higher yield, stability, and scalability, making them the preferred choice for the production of biopharmaceuticals such as monoclonal antibodies (Kunert and Reinhart 2016).

A key challenge still faced by the biopharmaceutical industry is the accumulation of lactate in cell culture. Lactate accumulation is a result of aerobic glycolysis, also known as the Warburg effect, which is the conversion of glucose to lactate despite the presence of oxygen. The Warburg effect is characteristic of CHO cells (Hartley et al. 2018), which typically secrete lactate during the exponential (growth) phase of cell culture, followed by a lactate switch where the accumulated lactate is then consumed by the cells. This lactate switch is a desired characteristic of cell culture and is linked to a healthy and more oxidative cell phenotype (Mulukutla et al. 2012), thereby encouraging a lactate switch, which can lead to higher titres and harvest viability.

Lactate secretion as a result of the Warburg effect is both inefficient and deleterious to cell cultures. The production of ATP through glycolysis only yields 2 ATP per glucose molecule, compared to the ∼32 ATP generated by oxidative phosphorylation (OXPHOS). Re‐routing the glucose‐derived pyruvate away from lactate production and into the TCA cycle and electron transport chain (ETC) would be a more effective utilisation of glucose and, in theory, could increase cell growth in the early stages of cell culture and boost final product titres.

As well as being an inefficient use of glucose, lactate itself has a negative impact on cell culture. When lactate levels rise to high concentrations, detrimental consequences on growth and productivity may arise (Buchsteiner et al. 2018; Fu et al. 2016; Hefzi et al. 2025; Lao and Toth 1997; Li et al. 2012; Torres et al. 2018). Lactate also has an indirect deleterious impact on cell culture, through the drop in pH upon accumulation in the extracellular environment. In a pH‐controlled bioreactor, lactate secretion lowers the pH below the setpoint, causing the addition of base. This base addition will raise osmolality and lead to inhibition of cell growth and productivity (Alhuthali et al. 2021; Romanova et al. 2022; Zhu et al. 2005).

Multiple explanations exist for CHO cells' preference for aerobic glycolysis, yet the underlying mechanisms are yet to be fully elucidated. Amongst these explanations are a rapid need for ATP generation (Liberti and Locasale 2016; Zheng 2012), synthesis of biomass precursors (Boroughs and Deberardinis 2015; Cairns et al. 2011; Heiden et al. 2009), impaired mitochondrial function (Heiden et al. 2009; Luengo et al. 2021; Sacco et al. 2023) and proteome constraints (Sánchez et al. 2017; Yeo et al. 2020). Following lactate accumulation in the exponential phase, multiple factors then influence the lactate switch, including bioreactor pH (Becker et al. 2019; Zalai et al. 2015), glucose and glutamine concentration (Ghorbaniaghdam et al. 2014; Wahrheit et al. 2014).

Another key driving force for aerobic glycolysis is the balance of redox cofactors NAD^+^ and NADH. Lactate production and secretion are controlled by a single reaction, lactate dehydrogenase (LDH), which reversibly converts pyruvate to lactate, simultaneously regenerating a molecule of NAD^+^ from NADH. In the exponential phase of cell culture, CHO cells consume large amounts of glucose, leading to high glycolytic fluxes, which consumes large amounts of NAD^+^ in the GAPDH reaction (Lunt and Vander Heiden 2011). In order to regenerate NAD^+^ to maintain glycolytic function, cells rely on LDH, and hence, lactate is secreted as a by‐product.

Increasing intracellular NAD^+^ concentrations may offer an effective strategy for reducing lactate secretion or even promoting lactate consumption. One approach involves adding NAD^+^ directly to the growth medium (Lee et al. 2023); however, this method is prohibitively expensive and results in reduced cell growth, rendering it unsuitable for large‐scale manufacturing. Alternative strategies include utilising NADH dehydrogenases (Diaz‐Ruiz et al. 2018; Kim et al. 2014; Talla et al. 2020), NADH oxidases (Geueke et al. 2003; Titov et al. 2016; Vemuri et al. 2007), or inhibiting NAD^+^ consuming enzymes such as Poly(ADP‐ribose) polymerases (PARPs) (Fang et al. 2014; Pirinen et al. 2014; Sarkar et al. 2023).

Another method to enhance NAD^+^ concentration is to target NAD^+^ biosynthesis pathways. There are three key pathways involved in NAD^+^ production: de novo biosynthesis from tryptophan, the Preiss–Handler pathway, and the salvage pathway (Denu 2007). Enzymes within these pathways can be manipulated to boost NAD^+^ levels. For instance, nicotinamide phosphoribosyl transferase (NAMPT) catalyses the conversion of nicotinamide (NAM) to NAD^+^ via nicotinamide mononucleotide (NMN). Intracellular NAD^+^ levels can be increased through the use of small‐molecule activators (Gardell et al. 2019; Yao et al. 2022) or NAMPT overexpression (Audrito et al. 2020; Wang et al. 2011). Since NAD^+^ acts as a feedback inhibitor of NAMPT (Burgos and Schramm 2008; Takahashi et al. 2010), activating NAMPT helps drive the equilibrium towards NAD^+^ production, enhancing its intracellular concentration beyond baseline levels. Other enzyme targets in NAD^+^ biosynthesis, such as NMNAT1 (Fang et al. 2022; Liang et al. 2015; Rossi et al. 2018), NAPRT (Baldassarri et al. 2023), NRK1 (Cercillieux et al. 2022), and NADS (Hashida et al. 2016), which have not yet been tested in CHO cells at the time of writing.

A more practical and cost‐effective strategy involves supplementing NAD^+^ biosynthesis precursors in CHO cell cultures. This has been tested in media formulations (Han et al. 2024), but not in feed design in fed‐batch cultures to improve lactate profiles. In mammals, tryptophan, niacin/nicotinic acid (NA), and niacinamide/nicotinamide (NAM) are dietary sources, while extensive recycling of NAD^+^ from NAM in the salvage pathway maintains cellular NAD^+^ levels (Liu et al. 2018). Several potential precursors, such as NAM, NA, nicotinamide riboside (NR), nicotinate mononucleotide (NAMN), nicotinate adenine dinucleotide (NAAD), quinolinic acid (QA), and tryptophan, are available for supplementation. The utilisation of these pathways is tissue‐ and cell line‐dependent (Liu et al. 2018), with prior transcriptomic data from the our CHO‐K1‐derived cell line reveals that the *de novo* biosynthesis pathway is inactive (*data not shown)*, making tryptophan and QA ineffective as supplements. Among the remaining options, cost considerations at large‐scale production favour NA and NAM, as they are inexpensive, unlike NR, NAMN, and NAAD, which are costly speciality chemicals and therefore not economically viable for large‐scale production. Consequently, NA and NAM were selected for this study.

## 2 Methodology

### 2.1 Cell‐Line and Fed‐Batch Conditions

A clonal cell line, transfected with a construct encoding glutamine synthetase (GS) and a human IgG1 monoclonal antibody, which was generated using the proprietary AstraZeneca CHO host cell line derived from CHO‐K1, was used in this study.

Cells were seeded at a density of 0.9×10^6^ cells mL^−1^ in proprietary media at an initial working volume of 14 mL in an Ambr®−15 system (Sartorius Stedim Biotech, Hertfordshire, UK). A two‐part feed was added, with the first feed occurring when cells reached a proprietary target density, and on even days going forward. Glucose was controlled at a defined range. Dissolved oxygen (DO) was controlled at 50%. The pH was controlled at 7.05 ± 0.1. When the upper deadband was reached, CO_2_ was gassed, and when the lower deadband was reached, base (sodium bicarbonate) was added. The temperature was controlled at 36.5°C.

NAM and NA (Sigma‐Aldrich, St. Louis, MO, USA) were added to the bioreactors from 1 M stock solutions. An initial shake flask screening study (data not shown) was conducted to determine optimal feed concentrations and timings. Based on these findings, the final feeding strategy included 2 mM and 5 mM additions into each feed day, as well as a single 10 mM addition on day 6. The stated NAM and NA concentrations represent the nominal bioreactor concentration immediately after feeding. The actual concentrations were not measured and may have exceeded these values if uptake was lower than the feeding rate. A combination of NAM & NA 2 mM supplementation on each feed day was also implemented.

### 2.2 Measurements

Extracellular glucose and lactate concentrations were measured with a YSI 2900D Biochemistry Analyzer (YSI, Yellow Springs, Ohio, USA). Glucose concentrations are normalised relative to the initial concentration of control vessels. Cell density and viability were measured by a Vi‐Cell XR (Beckman Coulter, Indianapolis, Indiana, USA).

Quantification of amino acid concentrations in supernatant samples was completed using the AccuTag system on a Waters Acquity ultraperformance liquid chromatography (UPLC) system (Waters, Elstree, UK) according to the manufacturer's instructions. Supernatant samples were collected on harvest day for mAb titre quantification, using a protein A high‐performance liquid chromatography (HPLC) on an Agilent 1260 Infinity series (Agilent Technologies, Santa Clara, CA, USA) by comparing peak size from each sample with a calibration curve.

Integral of viable cell density (IVCD), in 10^6^cells h mL^−1^ was calculated according to Equation 1.

(1) $${\mathrm{IVCD}}_{t}={\mathrm{IVCD}}_{t-1}+\frac{\left({\mathrm{VCD}}_{t}+{\mathrm{VCD}}_{t-1}\right)*∆t}{2}$$

Where ${\mathrm{VCD}}_{t}$ is the VCD at time t in 10^6^cells mL^−1^, ∆t is the time difference in hours.

Yield of lactate on glucose, $Y_{\mathrm{lac}/\mathrm{glc}}$, was calculated using Equation 2:

(2) $$Y_{\frac{\mathrm{lac}}{\mathrm{glc}}}=-\frac{q_{\mathrm{lac}}}{q_{\mathrm{glc}}}$$

Cell‐specific productivity at time t, ${\mathrm{qP}}_{t}$, in pg cell^−1^ h^−1^ was calculated using Equation 3:

(3) $${\mathrm{qP}}_{t}=\frac{{\mathrm{titre}}_{t}}{{\mathrm{IVCD}}_{t}}$$

Statistical analysis on $Y_{\mathrm{lac}/\mathrm{glc}}$ and ${\mathrm{qP}}_{t}$ to compare each feeding strategy with the control condition was performed using an unpaired two‐tailed *t*‐test in Microsoft Excel.

### 2.3 Glycan Profiling

UPLC Glycan Profiling of 2‐Aminobenzamide Labelled N‐Linked Glycans: Briefly, the monoclonal antibody was digested with Peptide‐N‐Glycosidase F (PNGase F) in 50 mM Tris‐HCl buffer, pH 7.8, at 37°C for 16 h to release N‐glycans from the monoclonal antibody. The released glycans were labelled with 2‐Aminobenzamide (2‐AB) by reductive amination. The labelled glycans were purified using a GlykoClean S Plus cartridge and separated by UPLC on an Acquity UPLC BEH Glycoprotein column using a gradient formed by Ammonium Formate and Acetonitrile mobile phases.

### 2.4 Transcriptomic Data

RNA was extracted from cell pellets containing 1×10^7^ cells, in accordance with the manufacturer's protocol of a RNeasy Mini Kit (Qiagen, Manchester, UK). 1×10^7^ cells were taken from bioreactors with varying experimental conditions on day 6 and stored using RNAlater (Thermo Fisher Scientific, Waltham, MA, USA). Total RNA was then extracted with the RNeasy Mini Kit according to the manufacturer's instructions. RNA quality was checked using a Bioanalyzer (Agilent, Santa Clara, CA, USA). Library preparation was conducted utilising a TruSeq Stranded Total RNA Sample Preparation (Illumina, San Diego, CA, USA). Sequencing was performed at the Next Generation Sequencing Facility at the University of Leeds on a NextSeq. 2000 sequencer (Illumina, San Diego, CA, USA).

Fastp (Chen et al. 2018), version 0.23.4, was used to trim PCR and sequencing adaptors and filter low‐quality read pairs. Trimmed and QC‐filtered reads were then used by Salmon (Patro et al. 2017), version 1.10.0, with the selective alignment strategy (Srivastava et al. 2020) to quantify the expression of transcripts, annotated in Ensembl, version 93 (Howe et al. 2021), with the addition of the extra transgenes. The Bioconductor package txtimport (Soneson et al. 2016) has been used to summarise transcript expression in terms of gene expression.

Differential gene expression (DGE) analysis was conducted using DESeq. 2 (Anders and Huber 2010) in R version 4.4.2. (Genes with expression less than 1 transcript per million (TPM) in each sample were filtered from the results. A gene was considered differentially expressed if the adjusted *p* value (*p*_adj_) was less than 0.01 and the absolute value of the Log2 fold change (Log2FC) was above 0.3. As RNA‐Seq data was taken on day 6, before the single‐dose feeds were added, there were 9 control experiments, 6 experiments with NAM feeding (2 mM and 5 mM feeds) and 6 with NA feeding (2 mM and 5 mM feeds) for the purposes of DGE analysis. DESeq. 2 analysis separating the 2 mM and 5 mM NAM feeds revealed similar differential expression between these sets and control (Figure S4), meaning they were combined together for DGE.

Functional gene enrichment was carried out using Gene Set Enrichment Analysis (GSEA) (Subramanian et al. 2007) using the GSEApy package (Fang et al. 2023) in Python. GSEA was performed using the WikiPathways 2024 Human database (Agrawal et al. 2024), with a pre‐ranked approach. A minimum gene set size of 3 and a maximum of 100 were used, with 1000 permutations. Pathways with a False Discovery Rate (FDR) *q*‐value below 0.05 were considered significant.

Principal component analysis (PCA) was performed using scikit‐learn (Pedregosa et al. 2011) in Python. Log₂(TPM + 1) expression values were standardised with StandardScaler, and PCA was conducted with “n_components=2”. The first two components were used to visualise variance between samples.

## 3 Results and Discussion

### 3.1 Nicotinamide Feed Supplementation Reverses the Warburg Effect

**Figure 1:**
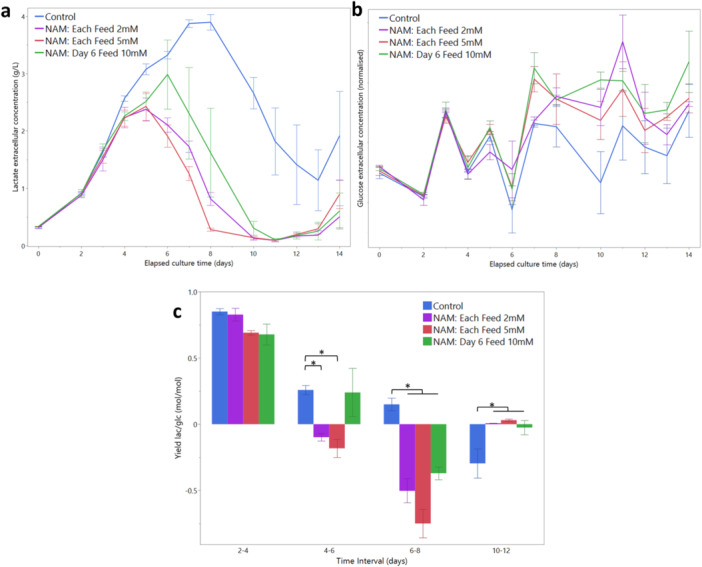
(a) Lactate concentration, (b) glucose concentration and (c) yield of lactate on glucose ($Y_{\mathrm{lac}/\mathrm{glc}}$) for the NAM‐fed bioreactors. Error bars indicate standard error for the Ambr‐15 vessel triplicates (*n* = 3). Asterisk indicates statistical difference from control (*t*‐test *p* value < 0.05). Glucose concentrations were normalised.

Three NAM feed supplementation strategies were tested in triplicate using an Ambr‐15 bioreactor system. As outlined in Table 1, these included two conditions with NAM feeding on each feed day at different concentrations (2 mM and 5 mM bioreactor concentration), and a single‐dose NAM feed on day 6 at 10 mM bioreactor concentration. Figure 1 illustrates the effects of these NAM feeding strategies on extracellular lactate and glucose concentrations, as well as the lactate yield on glucose.

**Table 1:** Summary of feeding strategies in Ambr‐15. Each strategy was tested in triplicate.

Feed strategy	Feed description
Control	Platform proprietary AstraZeneca feed.
NAM: Each Feed 2 mM	Nicotinamide is added to each feed day to achieve a nominal 2 mM bioreactor concentration.
NAM: Each Feed 5 mM	Nicotinamide is added to each feed day to achieve a nominal 5 mM bioreactor concentration.
NAM: Day 6 Feed 10 mM	Single dose nicotinamide feed at day 6 to achieve a nominal 10 mM bioreactor concentration.
NA: Each Feed 2 mM	Nicotinic acid is added to each feed day to achieve a nominal 2 mM bioreactor concentration.
NA: Each Feed 5 mM	Nicotinic acid is added to each feed day to achieve a nominal 5 mM bioreactor concentration.
NA: Day 6 Feed 10 mM	Single dose nicotinic acid feed at day 6 to achieve a nominal 10 mM bioreactor concentration.
NAM & NA: Each Feed	Nicotinamide and nicotinic acid is added to each feed day to achieve a nominal 2 mM bioreactor concentration of both supplements.

Figure 1a demonstrates that NAM feed supplementation significantly reduced the average peak lactate concentration, lowering it from an average of 4.0 g L^−1^ in the control triplicates to 2.4 g L^−1^ in the 2 mM and 5 mM NAM‐fed cultures (*p* = 1.5×10^‐4^), a 40% decrease in peak lactate concentration. As well as decreasing peak lactate, NAM feeding induced a shift to lactate consumption, with the lactate shift occurring on day 5 for both the 2 mM and 5 mM fed cultures, compared to day 8 in the control cultures.

Lactate concentration declined rapidly following this metabolic switch, indicating high rates of lactate consumption under these conditions. This effect is most pronounced for the single‐dose 10 mM NAM feed, where lactate levels drop sharply after day 6. Figure 1c further demonstrates this, showing how $Y_{\mathrm{lac}/\mathrm{glc}}$ turned negative (indicating lactate consumption) after NAM supplementation, while remaining positive in the control condition.

This early switch to lactate consumption is attributed to the conversion of NAM to NAD^+^ via the salvage pathway, mediated by NAMPT through NMN, leading to increased NAD⁺ concentrations (Han et al. 2024). The resulting elevated NAD^+^/NADH ratio strongly favours NAD^+^ consumption, converting lactate to pyruvate via LDH while generating NADH. Transcriptomic analysis on day 6 of cell culture, discussed further in Section 3.4, supports this metabolic shift, revealing significant upregulation of mitochondrial ETC genes (*Nd1*, *Nd2*, *Nd3*, *Nd4*, *Nd4l*, *Nd5*, *Cytb*, and *Apt6*), which suggests a transition towards OXPHOS. Additionally, the downregulation of *Hk2* indicates reduced glycolytic flux, reinforcing the observed decrease in glucose uptake. These transcriptomic changes align with the metabolic shift away from the Warburg effect.

In NAM‐fed conditions, lactate serves as an alternative carbon source to glucose. As shown in Figure 1b, glucose uptake is reduced in the NAM‐fed vessels, with lactate instead being utilised as a carbon source. This reduced glucose uptake leads to a consistent net flow of carbon toward pyruvate across all conditions, reflecting the highly regulated pathways downstream of pyruvate, such as the TCA cycle (Chong et al. 2010).

An additional factor contributing to the shift from glucose to lactate as a carbon source may be the regulatory impact of altered NAD^+^ metabolism. NAD^+^ is a crucial substrate for regulatory proteins like SIRTs and PARPs (D'Amours et al. 1999; Imai et al. 2000), and the increased NAD^+^ concentrations in NAM‐fed conditions could impact glycolysis. For instance, PARP1 is known to inhibit hexokinase 1 (Fouquerel et al. 2014), suggesting that the heightened activity of these regulatory proteins may restrict glucose uptake. Additionally, NAM itself can inhibit SIRT activity (Bitterman et al. 2002; North and Verdin 2004). SIRTs break down NAD^+^ into NAM during the deacetylation process and are auto‐inhibited by NAM. This inhibition is especially significant for SIRT1 (Bitterman et al. 2002), which plays a key role in regulating glycolysis (Pinho et al. 2016; Rodgers et al. 2005). Therefore, the intracellular accumulation of NAM might block SIRT1 activity, potentially downregulating glycolysis and leading to reduced glucose uptake. SIRTs 1‐3 activity also inhibits hypoxia‐inducing factor‐1α (*Hif1a*) (Greer et al. 2012), which regulates cellular response to hypoxia, by promoting glycolysis‐related gene expression and suppressing OXPHOS. Further discussion and analysis of gene expression is found in Section 3.4.

### 3.2 High Nicotinamide Concentrations Attenuate Cell Growth

**Figure 2:**
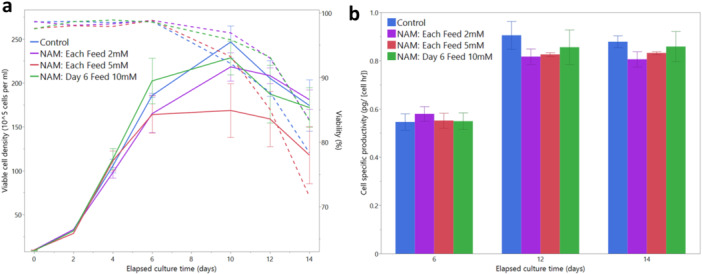
(a) Viable cell density (VCD), viability (dashed line) and (b) cell‐specific productivity for the recombinant production in the NAM‐fed conditions. Error bars indicate standard error for the triplicates (*n* = 3).

Supplementation with NAM positively affects lactate profiles, but at high concentrations, it suppresses cell growth. As illustrated in Figure 2a, a NAM feed concentration of 5 mM leads to a plateau in VCD starting from day 6 of culture and causes a more rapid decline in cell viability. The IVCD at harvest (day 14) for the control conditions averages at 5.3×10^12^ cell h mL^−1^, while it is 4.2×10^12^ cell h mL^−1^ for the 5 mM NAM feed, a 20% decrease. These effects are less pronounced under conditions of lower NAM feed concentrations (harvest IVCD at 2 mM and single dose feed is 4.9 and 5.2×10^12^ cell h mL^−1^, respectively), suggesting a trade‐off is needed between promoting a more oxidative state and affecting cell growth.

Several mechanisms may explain how NAM supplementation influences growth rates, and the lowered growth rate is supported by a downregulation of a host of growth‐related genes, which are discussed further in Section 3.4. One explanation is reduced glycolytic flux, resulting from the switch to lactate as a carbon source instead of glucose in the NAM‐fed conditions. This glycolytic suppression can decrease cell proliferation, as glycolytic intermediates are vital precursors for nucleotide, amino acid, and lipid synthesis (Horváthová et al. 2021; Jones and Bianchi 2015). With less glycolysis, fewer intermediates are available, compromising the synthesis of macromolecules essential for cell growth and division. For instance, glycolysis provides precursors like 3‐phosphoglycerate, which is converted to serine and subsequently to glycine and cysteine, essential amino acids (Amelio et al. 2014). However, it has been shown that the high glycolytic fluxes are not essential to obtain biomass precursors, and a wild‐type growth rate can be achieved in Warburg‐null CHO cell lines (Hefzi et al. 2025).

Reduced glycolytic flux may contribute to an energy deficit, as the conversion of lactate to pyruvate via LDH does not generate ATP, unlike glycolysis, which produces two ATP molecules per glucose. However, the anticipated increase in OXPHOS capacity (see Section 3.4) may help offset this energy shortfall by enhancing ATP production through more efficient mitochondrial respiration. Furthermore, the synthesis of NAD^+^ from NAM through the salvage pathway imposes an additional ATP burden, requiring two ATP molecules per NAD^+^ molecule synthesised (Xie et al. 2020). Han et al. (2024) demonstrated that cells treated with NAD^+^ precursors exhibited lower ATP levels than untreated cells, reinforcing the idea that NAD^+^ biosynthesis increases cellular energy demand. Beyond ATP consumption, NAD^+^ synthesis from NAM consumes phosphoribosyl pyrophosphate (PRPP) as a key precursor, increasing the demand for nucleotides. The combined strain on ATP and nucleotide pools could explain the observed reduction in cell growth under high NAM concentrations.

Elevated NAD^+^ from NAM feeding also influences the NAD^+^ dependent regulatory proteins, such as SIRTs and PARPs, as discussed in Section 3.1, which may have negative effects on cell growth. Excessive PARP activity has been linked to DNA damage, genomic instability, and impaired cell proliferation (Kang et al. 2022). SIRTs, crucial for metabolic regulation, can be affected both by increased NAD^+^ and NAM itself. While NAD^+^ serves as a substrate for SIRT activation, NAM acts as a feedback inhibitor (Bitterman et al. 2002; North and Verdin 2004). The concurrent rise in both activator and inhibitor levels could create unpredictable metabolic effects. Overactivity of SIRTs like SIRT1 and SIRT6, which deacetylate histones, could result in significant epigenetic changes that suppress genes required for growth and division. For instance, SIRT1 has been shown to inhibit the mTOR pathway, a key regulator of cell growth (Ghosh et al. 2010). Overactive mitochondrial SIRTs (SIRT3‐5) may increase oxidative stress by enhancing mitochondrial metabolism, while NAM‐induced inhibition of SIRTs may disrupt metabolic regulation, reducing ATP production and the availability of biosynthetic precursors necessary for growth. Additionally, SIRT1's interaction with AMPK, a vital energy sensor, may be compromised with lowered SIRT1 activity, leading to metabolic dysregulation (Price et al. 2012).

Although high NAM concentrations inhibit growth, cell‐specific productivity is not significantly affected (Figure 2b), indicating that productivity is not reduced as drastically as growth rates. Since the impact of NAM on growth is dose‐dependent, it is crucial to find a balance between its benefits and drawbacks. Implementing NAM supplementation in a platform process should involve optimising concentrations to avoid adverse effects on growth or titre (Figure S1).

### 3.3 Nicotinic Acid Supplementation Leads to Lactate Secretion Spiral

**Figure 3:**
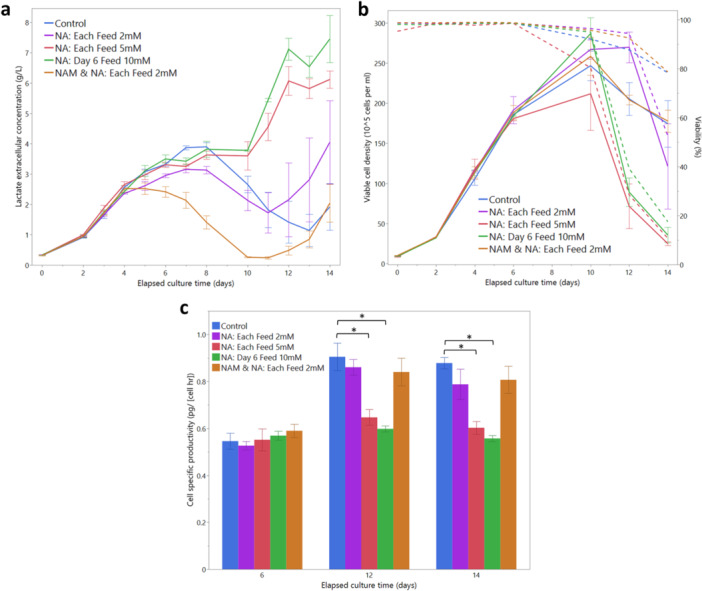
(a) Lactate concentration, (b) viable cell density (VCD), viability (dashed line) and (c) cell‐specific productivity (qP) for the NA fed‐conditions. Error bars indicate standard error for the triplicates. Asterisk indicates statistical difference from control (*p* value < 0.05).

NA feed supplementation did not yield the same beneficial outcomes as NAM. As shown in Figure 3a, NA‐fed cultures initially maintained lactate levels comparable to the control but experienced a sharp increase in lactate concentration in the final days of culture. This lactate spike coincided with a decline in VCD and cell viability (Figure 3b). The excessive lactate secretion led to a decrease in pH, necessitating the addition of large volumes of base toward the end of the culture period. This base addition, in turn, triggered further lactate secretion, creating a ‘lactate secretion spiral’ observed in the NA‐fed conditions.

Even though culture vessels were pH‐controlled, and the NA stock solution was buffered to neutral pH to minimise any direct impact on culture pH, the uptake of NA into cells may have caused a significant drop in intracellular pH beyond the capacity of acid‐base transporters to regulate (Doyen et al. 2022). This intracellular acidification disrupts the mitochondrial proton gradient (Poburko et al. 2011), inhibits critical enzymes (Jubrias et al. 2003), and alters proton‐dependent transport across the mitochondrial membrane (Selivanov et al. 2008).

The resulting lactate secretion sets off a secretion spiral in the following way: base is added to maintain the pH set point, which increases the osmolality beyond tolerable levels. Elevated osmolality further impairs OXPHOS (Lee et al. 2003; Pan et al. 2019; Romanova et al. 2022), prompting even more lactate secretion and further base addition.

An additional complicating factor is the cellular uptake mechanism of NA. NA is transported into cells via the sodium‐dependent MCT (SMCT) located on the plasma membrane (Bongarzone et al. 2020; Gopal et al. 2007, 2005; Yanase et al. 2008), which co‐transports NA with one sodium ion. Since the base used in the culture is sodium bicarbonate, the increasing sodium concentration gradient from base addition enhances NA uptake via the SMCT. This further uptake is expected to exacerbate intracellular acidification, leading to more lactate secretion through MCT to counteract the pH imbalance. However, this response may be insufficient to maintain homoeostasis, resulting in the observed decrease in cell viability.

### 3.4 Differential Gene Expression Analysis Confirms Switch to Oxidative Metabolism

**Figure 4:**
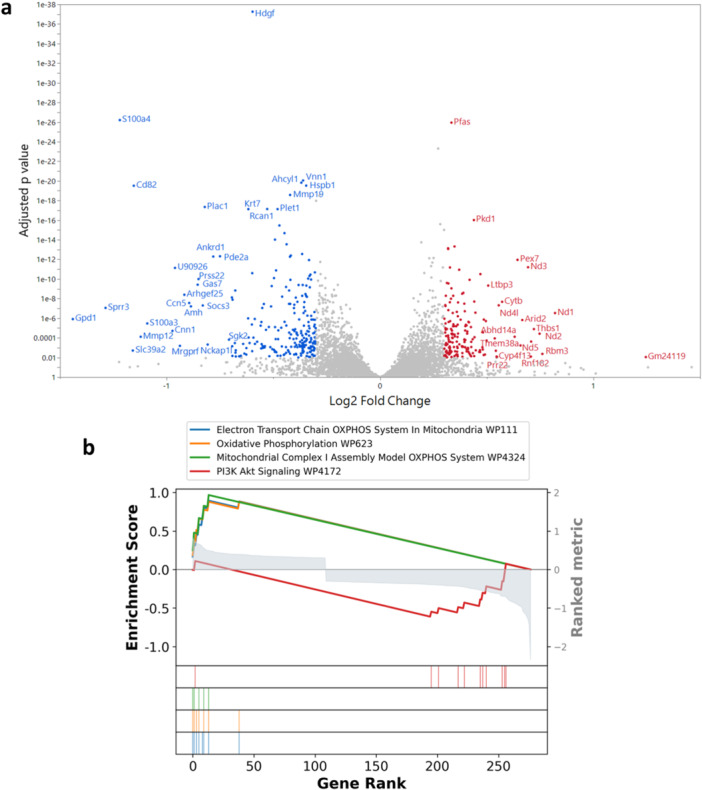
(a) Volcano plot from differential gene expression (DGE) analysis between control (*n* = 9) and NAM‐fed (*n* = 6) conditions at day 6. Upregulated genes (113) are shown in red, downregulated genes (186) in blue. Cutoff for differential expression is *p*_adj_ < 0.01 and abs(Log2 FC) > 0.3. (b) GSEA plot showing enriched pathways from the DGE analysis.

**Figure 5:**
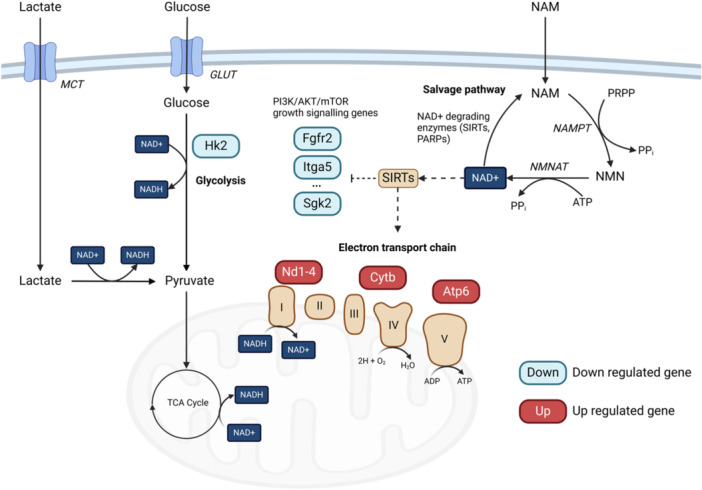
Summary of the impact of NAM feeding. NAM is transported into the cell, converted to NMN and finally NAD^+^ in the salvage pathway. NAD^+^ impacts the Sirtuin class of regulatory proteins, impacting energy metabolism, including the electron transport chain and growth signalling genes, which leads to a lower growth rate. As a result of increased NAD^+^, lactate is consumed alongside glucose, regenerating NADH in the LDH reaction. Glucose uptake and glycolysis are downregulated, while mitochondrial ETC genes are upregulated as cells rely on mitochondrial respiration for ATP. Red genes are upregulated, blue are downregulated, dashed lines indicate activation, and blocked lines indicate inhibition. Created with BioRender.com.

Transcriptomic analysis reveals a shift from glycolytic to oxidative metabolism, which provides further explanation for the reverse in the Warburg effect in NAM‐fed cultures. Day 6 represents a crucial point in culture, as the control conditions were still secreting lactate, while the NAM‐fed cells had just undergone the lactate switch. DGE analysis between control and NAM‐fed using DESeq. 2 (Section 3.3) revealed 113 upregulated genes and 186 downregulated genes. Figure 4 displays a volcano plot of this DGE analysis, while Figure 5 (below) summarises the key impacts of NAM feeding on CHO cell metabolism. The full list of up‐ and downregulated genes is available in Table S1.

A key observation is the upregulation of multiple genes in the mitochondrial ETC, particularly *Nd1*, *Nd2*, *Nd3*, *Nd4*, *Nd4l*, *Nd5*, *Cytb*, and *Apt6*, which encode subunits of mitochondrial Complexes I, III, and V. This strongly suggests an increase in OXPHOS activity in NAM‐fed cultures, consistent with the observed reduction in lactate secretion and the shift away from aerobic glycolysis.

One of the downregulated genes with the largest fold change is *Gpd1* (glycerol‐3‐phosphate dehydrogenase). *Gpd1* is part of the glycerol‐3‐phosphate shuttle, transferring NADH electrons to mitochondria and replenishing cytosolic NAD^+^. But with elevated NAD^+^ levels and reduced glycolysis, there is less NADH to be transported into the mitochondria, and this gene is significantly downregulated.

The high expression of *Pfas* (phosphoribosylformylglycinamidine synthase) in NAM‐fed conditions is likely driven by the increased demand for nucleotides in NAM‐fed conditions. Since NAM is converted to NAD^+^ via the salvage pathway, this process consumes both ATP and PRPP, a key substrate in de novo purine biosynthesis. To compensate, cells upregulate *Pfas*, a crucial enzyme in purine metabolism, to meet purine demand. The increased flux through this reaction may also explain the high glutamine consumption rates and accumulation of glutamate in NAM‐fed conditions (Figure S2), as *Pfas*, and other reactions in purine synthesis use glutamine as a nitrogen donor.

Several groups of downregulated genes provide insight into the reduced cell growth observed at high NAM concentrations. These include downregulation of regulatory genes in growth signalling, such as WNT, MAPK and PI3K/AKT/mTOR pathway (*Sgk2*, *Tlr2*, *Angpt4*, *Il6*, *Lamc2*, *Creb3l1*, *Thbs2*, *Fgfr2*, *Dusp14, Fhl2, Socs3, Wnt4, Ccn5*), cell cycle and proliferation (*Hdgf, S100a4, Syne2, Kntc1, Spdl1*) and extracellular matrix and adhesion (*Mmp9, Mmp12, Pdpn, Col5a2, Itga5*). Growth signalling, particularly the WNT pathway, is strongly linked to aerobic glycolysis (Pate et al. 2014; Thompson 2014; Vallée et al. 2021), and the shift from Warburg metabolism could impact these regulatory pathways to lower cell proliferation. As discussed in Sections 3.1 and 3.2, NAD^+^ dependent (and NAM inhibited) SIRTs may be impacting these regulatory processes, which have been shown to interact with PI3K/AKT/mTOR pathway to reduce cell growth (Fan et al. 2023; Ghosh et al. 2010; Sadria and Layton 2021).

Functional gene enrichment analysis using GSEA (Section 3.3 and Table S2) further supports these metabolic changes (Figure 4b). Of the four altered pathways, three positively enriched pathways were related to oxidative phosphorylation and mitochondrial ETC activity, while the negatively enriched pathway was linked to PI3K/AKT/mTOR signalling. These findings reinforce the metabolic shift towards OXPHOS and the suppression of growth‐associated signalling pathways in NAM‐fed cultures. While the metabolic and transcriptomic data are consistent with altered NAD^+^/NADH balance, direct quantification of intracellular NAD+ and NADH will be required to confirm this mechanism.

DGE analysis was applied to NA feed supplementation. NA‐fed gene expressions were much more similar to the control than NAM‐fed conditions. This is evident in the PCA plot of gene expression (Figure S3), as well as by comparing process data on day 6 in Section 3.4. Consequently, only one gene was differentially expressed, which was the downregulation of *Ogdh* (oxoglutarate dehydrogenase). *Ogdh* is a critical TCA cycle gene as well as a mitochondrial redox sensor and regulator, and its downregulation represents an early response to oxidative stress (Chang et al. 2022; McLain et al. 2011), suggesting that NA feeding may have initiated early signs of cellular stress. However, since these gene expression results were collected on day 6, prior to the significant drop in viability observed in NA‐fed cultures, the later decline in cell health is not fully explained.

### 3.5 NAM Supplementation Increases Fc Galactosylation Without Altering Other Major Glycan Features

**Figure 6:**
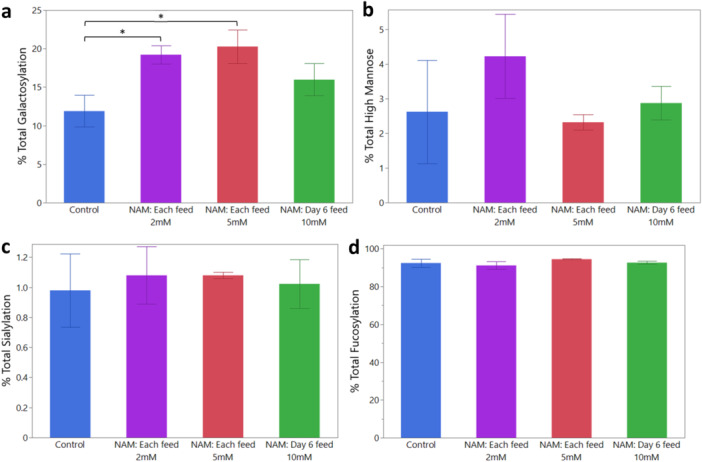
Comparison of Fc N‐glycan composition across control and NAM‐supplemented cultures at harvest (day 14). Shown are the relative abundances (%) of (a) galactosylated species, (b) high mannose species, (c) total sialylated species and (d) core‐fucosylated species.

The metabolic reprogramming induced by NAM supplementation was accompanied by a modest increase in Fc terminal galactosylation, while other glycan features remained largely unchanged (Figure 6). Total galactosylation increased from 11.9% in the control to 19.2% (*p* = *0.006*) and 20.3% (*p* = *0.009*) in the 2 mM and 5 mM NAM each‐feed conditions, respectively. The single 10 mM day 6 feed produced a more moderate increase to 16.0% (*p* = *0.073*). In contrast, total sialylation (1.0–1.1%) and total fucosylation (91.2–94.5%) remained largely unchanged, and high mannose species were comparable to control except for a modest increase at 2 mM NAM (4.2%), which is not statistically significant nor observed at 5 mM NAM (2.3%), suggesting that the increase at 2 mM may not represent a systematic processing defect. The full N‐glycan distribution for all samples is reported in Table S3.

The increase in galactosylation is consistent with several process‐level effects of NAM supplementation. First, the lower cumulative lactate production reduces base addition requirements and therefore limits hyperosmotic stress. Elevated osmolality in CHO is known to impair Golgi function, reduce galactosyltransferase efficiency, and shorten glycan maturation time (Alhuthali et al. 2021; Becker et al. 2019; Lee et al. 2003). Second, higher NAM concentrations attenuated cell growth, which can reduce secretory flux and extend ER/Golgi residence time, allowing more complete terminal processing (Fisher et al. 2019; Nguyen et al. 2021). Finally, lower lactate likely mitigates intracellular acidification, promoting more favourable Golgi enzyme kinetics and glycosyltransferase activity (Lao and Toth 1997; Reddy et al. 2025).

## 4 Conclusion

In this study, NAD^+^ precursors NAM and NA were supplemented in a fed‐batch CHO cell culture. NAM supplementation reduces peak lactate by 40% and reverses the Warburg effect to induce the lactate shift 3 days earlier than control conditions. Transcriptomic data reveal NAM promotes a shift from glycolytic to oxidative metabolism, with an upregulation of key mitochondrial ETC genes. These positive impacts are theorised to be caused by an increase in intracellular NAD^+^ concentrations due to synthesis from NAM in the salvage pathway.

NAM supplementation presents a simple and cost‐effective strategy to alleviate lactate accumulation in upstream bioprocessing. Incorporating NAM into feed formulations could counter rising lactate levels and mitigate their negative effects on cell viability and product quality. By promoting OXPHOS and moderating cell proliferation, NAM may also shift cultures toward a more energy‐ and nutrient‐efficient metabolic state. In perfusion cultures, where media consumption is a major cost driver, limiting excessive biomass accumulation may reduce nutrient waste and direct more resources toward recombinant protein production. Furthermore, preventing lactate secretion reduces the need for base addition to maintain bioreactor pH, limiting hyperosmotic stress and enabling more aggressive feeding strategies. These effects may be particularly beneficial in intensified fed‐batch processes, where nutrient demand is high but the risk of hyperosmotic stress and runaway lactate accumulation is increased. In contrast, NA supplementation did not produce similar metabolic benefits and instead triggered late‐stage lactate accumulation with reduced cell viability. The downregulation of *Ogdh*, a key mitochondrial redox sensor, suggested early signs of cellular stress that may contribute to the later decline in culture performance.

NAM could be incorporated into bioreactor feeds during process development, particularly for cell lines prone to lactate accumulation, or used as a corrective strategy in cases of runaway lactate during large‐scale production. The approach may be especially relevant for perfusion and intensified fed‐batch processes where lactate control and osmolality management are critical. However, this strategy has so far been demonstrated in a single cell line, and further validation will be required to establish its generalisability across CHO variants and production platforms. In the present study, NAM supplementation primarily improved lactate control and promoted a more oxidative phenotype. Although these effects did not increase biomass or titre under the tested conditions, improved lactate management may enhance process robustness and support productivity gains following optimisation of concentration and timing.

High concentrations of NAM attenuated cell growth, with DGE and functional enrichment revealing downregulation of growth regulatory genes, particularly within the PI3K/AKT/mTOR pathway. This likely reflects changes in NAD^+^‐dependent SIRT activity, reduced glycolytic flux, and increased energetic and nucleoside demands associated with NAD^+^ synthesis. Despite this growth attenuation, cell‐specific productivity remained unaffected, indicating that controlled NAM feeding can optimise lactate metabolism without compromising recombinant protein production.

Direct supplementation of NAM in basal medium was previously assessed across 100 µM ‐ 5 mM. Under those conditions, no meaningful impact on lactate metabolism was detected, and growth suppression was observed at the higher end of this range (data not shown). These observations motivated a feed‐based strategy to achieve substantially higher NAM exposure during the production phase while minimising early growth inhibition. However, the present study did not systematically compare equivalent total NAM doses delivered via basal medium versus feed. Although the composition of the proprietary AstraZeneca basal medium cannot be disclosed, commercially available CHO basal media typically contain NAM at approximately ~50 µM, and standard feed formulations contribute only low micromolar concentrations (generally < 5 µM at the vessel level). By contrast, the supplementation levels evaluated in this study (2–5 mM per feed or a single 10 mM addition) are roughly three orders of magnitude above these background levels. Collectively, the evidence indicates that simply increasing NAM in the basal medium is unlikely to reproduce the benefits observed with feed‐based delivery, which enables high NAM exposure at the appropriate process stage without compromising early growth.

A NAM supplementation strategy requires careful optimisation of both concentration and timing, as delivery mode may influence the balance between Warburg modulation and effects on cell growth and metabolism. Additional nucleosides may also need to be supplemented alongside NAM to support NAD^+^ synthesis without draining cellular resources. Metabolomics analysis further revealed significantly increased consumption of glutamine and alanine, with reduced glutamate, glycine, and aspartate uptake (Figure S2). Adjusting amino acid ratios may therefore assist cellular adaptation to a high‐NAD^+^ metabolic environment during NAM feeding.

## Author Contributions

Conceptualisation, methodology, formal analysis, writing, reviewing, editing: James Morrissey. Conceptualisation, methodology, investigation, supervision, funding acquisition, resources, reviewing, editing: Ayca Cankorur‐Cetinkaya. Methodology, investigation, reviewing, editing: Annie J. Harwood‐Stamper, Ryte Poskute, Kasia Kozakowska‐McDonnell. Conceptualisation, supervision, funding acquisition, reviewing, editing: Jonathan Welsh, Cleo Kontoravdi.

## Conflicts of Interest

Ayca Cankorur‐Cetinkaya, Luigi Grassi, Annie J. Harwood‐Stamper, James Morrissey, Ryte Poskute and Kasia Kozakowska‐McDonnell are employees of AstraZeneca.

## Supporting information

Supporting File 1

## Data Availability

The data that support the findings of this study are available from the corresponding author upon reasonable request.
